# Hyperspectral and Fluorescence Imaging Approaches for Nondestructive Detection of Rice Chlorophyll

**DOI:** 10.3390/plants13091270

**Published:** 2024-05-03

**Authors:** Ju Zhou, Feiyi Li, Xinwu Wang, Heng Yin, Wenjing Zhang, Jiaoyang Du, Haibo Pu

**Affiliations:** 1 College of Information Engineering, Sichuan Agricultural University, Ya’an 625000, China; 201803805@stu.sicau.edu.cn (J.Z.); 202005483@stu.sicau.edu.cn (F.L.); 202205894@stu.sicau.edu.cn (H.Y.); 202205963@stu.sicau.edu.cn (W.Z.); 2 College of Mechanical and Electrical Engineering, Sichuan Agricultural University, Ya’an 625000, China; wangxinwu2023@163.com; 3 Forge Business School, Chongqing Yitong University, He’chuan 401520, China; 19122317838@163.com

**Keywords:** hyperspectral, fluorescence spectrum, rice, non-destructive testing

## Abstract

Estimating and monitoring chlorophyll content is a critical step in crop spectral image analysis. The quick, non-destructive assessment of chlorophyll content in rice leaves can optimize nitrogen fertilization, benefit the environment and economy, and improve rice production management and quality. In this research, spectral analysis of rice leaves is performed using hyperspectral and fluorescence spectroscopy for the detection of chlorophyll content in rice leaves. This study generated ninety experimental spectral datasets by collecting rice leaf samples from a farm in Sichuan Province, China. By implementing a feature extraction algorithm, this study compresses redundant spectral bands and subsequently constructs machine learning models to reveal latent correlations among the extracted features. The prediction capabilities of six feature extraction methods and four machine learning algorithms in two types of spectral data are examined, and an accurate method of predicting chlorophyll concentration in rice leaves was devised. The IVSO-IVISSA (Iteratively Variable Subset Optimization–Interval Variable Iterative Space Shrinkage Approach) quadratic feature combination approach, based on fluorescence spectrum data, has the best prediction performance among the CNN+LSTM (Convolutional Neural Network Long Short-Term Memory) algorithms, with corresponding RMSE-Train (Root Mean Squared Error), RMSE-Test, and RPD (Ratio of standard deviation of the validation set to standard error of prediction) indexes of 0.26, 0.29, and 2.64, respectively. We demonstrated in this study that hyperspectral and fluorescence spectroscopy, when analyzed with feature extraction and machine learning methods, provide a new avenue for rapid and non-destructive crop health monitoring, which is critical to the advancement of smart and precision agriculture.

## 1. Introduction

Rice is a vital human food crop with a long history of production and consumption. Rice is consumed as a staple food by more than half of the world’s population because it is extremely adaptable to varied environments, making it an essential staple crop worldwide [1]. Rice production is the world’s third highest in terms of food production, only after corn and wheat [2]. The primary yield-limiting factors in rice farming are water and fertilizers, and a lack of fertilizers will cause changes in external morphology and internal structure, such as the thickness and color of leaves, which will result in variations in the reflectance properties of rice leaves and the spectrum of tree crowns [3]. Traditional indicators of crop growth include chlorophyll content and nitrogen content. The main methods of measurement are manual measurement, indoor chemical analysis [4], and the rapid determination of leaf chlorophyll by SPAD (Soil and Plant Analyzer Develotrnent) device. Molina L et al. used atomic absorption spectrometry to determine the content of heavy metals in rice [5]. Atomic absorption spectrometry can be used to assess magnesium content and, consequently, chlorophyll content indirectly. The concentration of chlorophyll in the extract can also be calculated using a spectrophotometer to determine the absorbance value of the chlorophyll extract at the maximum absorption wavelength [6]. Traditional detection methods are laborious and frequently require a significant amount of time and work, which hinders the development of plant growth monitoring and precise management to some extent. An ultra-portable SPAD assessment system for leaf SPAD distribution analysis was proposed by Tan LH et al. [7], which was used to further calculate leaf chlorophyll content. The collection of plant growth information by traditional analysis methods has gradually evolved remote sensing technology and its application in the field of agriculture, particularly with the advent of hyperspectral remote sensing technology.

Chlorophyll is the principal pigment involved in photosynthesis in plants, with its concentration directly affecting a plant’s capacity to absorb light energy; therefore, monitoring chlorophyll levels in plants is of paramount importance [8]. A deficiency in chlorophyll within rice leaves can arise from various factors, such as a lack of nitrogen, a deficiency in essential trace nutrients, or unsuitable soil acidity. Timely interventions can prevent yield losses caused by insufficient chlorophyll. Upon detecting a deficiency in chlorophyll, it is imperative to promptly analyze the cause and take corrective measures such as adjusting irrigation management, conducting soil tests, and swiftly replenishing nutrients. Given that chlorophyll content affects the spectral reflectance of rice, particularly within the visible light spectrum, reflectance spectra can be utilized to estimate chlorophyll content. Kandpal, K.C. and colleagues compared various methods of chlorophyll detection in leaves under laboratory and field conditions, concluding that Arnon’s spectrophotometric method is most suitable for laboratory settings, while machine learning methods are widely employed in chlorophyll detection tasks based on hyperspectral data [9]. Zhao, J.W. employed hyperspectral technology to detect chlorophyll content in tea leaves, with the constructed MSAVI2 model achieving an optimal result of RMSE = 8.60 on the test set, proving the viability of measuring chlorophyll content in tea leaves through hyperspectral imaging technology [10]. Jang, S.H. established a combined model of stepwise multiple linear regression and partial least squares to measure chlorophyll content in cucumber seedling leaves, identifying nine critical bands including those at 501 and 505 nm, enabling accurate and non-destructive detection of chlorophyll content in the leaves [11]. Yang, Y.C. and colleagues targeted 335 wheat varieties to establish a non-destructive model for detecting the SPAD value of wheat leaves, with experimental results indicating that the first-order reflectance at 549 nm and 735 nm had the strongest correlation with SPAD values. Cao, Y.L. et al. utilized drones equipped with spectral sensors to collect rice spectral images, successfully estimating chlorophyll content using a mathematical inversion model, thereby providing research ideas and data support for related research fields [12]. Feng, H. et al. developed an automatic spectral data analysis system using spectral imaging technology, discovering that chlorophyll, among four rice pigments, exhibited the strongest correlation in the 700–760 nm range [13].

Theoretical support exists for spectroscopic detection of chlorophyll concentration in rice leaves [14]. However, since different samples and experimental settings result in distinct chlorophyll sensitive bands, there is a need for a quick, non-destructive detection approach with broader applicability. As artificial intelligence technology advances, more scientists are employing machine learning to hyperspectral detection. Liu, H.H. et al. employed a hyperspectral imager on a drone to predict the chlorophyll content of a rice canopy by analyzing the obtained hyperspectral images [15]. Yang, Y.C. et al. proposed a more precise approach for non-destructive detection of chlorophyll using hyperspectral images and then studied the mechanism of photosynthesis in wheat drought tolerance [16]. Ruszczak, B. et al. examined 15 machine learning methods and chose the most effective model for detecting chlorophyll content in the hyperspectral data of rice leaves [17]. The utilization of machine learning algorithms aims to construct a non-invasive detection model. In the research presented herein, a system capable of rapidly and non-destructively determining the chlorophyll content in rice leaves through spectral data has been developed. With this model, spectral collection from rice leaves suffices to obtain the corresponding chlorophyll values, obviating the need for destructive measurement methods. The accurate detection of chlorophyll content can effectively detect the nutritional state of rice, which aids in the scientific management of agricultural production, fertilization, and grain yield.

Fluorescence is a powerful tool to study photosynthetic performance, especially when coupled with other noninvasive measurements such as absorption spectroscopy, gas analyses, and infrared thermometry. The use of chlorophyll fluorescence measurements to examine photosynthetic performance and stress in algae and plants is now widespread in physiological and ecophysiological studies [18]. Malenovsky, Z. posited that supplementing the measurement of vegetation physicochemical characteristics with fluorescence measurement methods, based on existing spatial platforms, can help eliminate errors and uncertainties in the interpretation of recent remote sensing data. Remote sensing of plant fluorescence signals can contribute to a better understanding of the photosynthesis process in vegetation [19]. Mishra, A. employed chlorophyll fluorescence imaging and compared eight classifiers and four feature selection methods, resulting in a species discrimination model with enhanced resolution efficiency [20]. Mattila, H. utilized a pulse-amplitude-modulated fluorescence camera imaging technique, which enabled the capture of fluorescence induction curve characteristics for each pixel in an image, achieving a 92.2% identification accuracy rate in the recognition of oat leaves [21]. Codrea, M.C. used a chlorophyll fluorescence kinetics imaging technique that allows for the simultaneous use of multiple fluorescence traits to determine photosynthetic mutants, facilitating rapid and non-destructive screening of these mutants [22]. Tyystjärvi, E., through the construction of a chlorophyll fluorescence fingerprint spectrum, successfully differentiated corn and barley from six weed species, extracting 17 features from fluorescence induction curves based on a neural network classifier with an accuracy rate ranging from 50.2% to 80.8% [23]. Fluorescence spectroscopy has demonstrated considerable promise in a host of applications, ranging from growth monitoring to the identification of diseases and stressors. Empirical evidence attests to the capability of fluorescence spectroscopy in pinpointing various stress factors affecting rice, including hydric stress, nutritional shortages, and pathogenic invasions. For example, research indicates that fluorescence metrics can exhibit marked variations under differing conditions of water scarcity, yielding invaluable data for the optimization of irrigation strategies [24]. In a similar vein, deficiencies in key nutrients such as nitrogen and phosphorus induce discernible changes in the fluorescence profiles of rice foliage, facilitating the prompt detection and rectification of these insufficiencies [25].

The utility of fluorescence spectroscopy has also been harnessed for the differentiation of rice cultivars [26]. Yang et al. advocated for the application of Laser-Induced Fluorescence (LIF) in conjunction with a multivariate analytical approach, incorporating Principal Component Analysis (PCA) and Support Vector Machine (SVM), to distinguish various paddy rice varieties [27]. Zhang et al. endeavored to delineate the correlation between the chlorophyll fluorescence spectra of rice and its growth dynamics, employing the PCA to facilitate the prognostication of rice development [28].

To achieve the accurate, rapid, and non-destructive detection of chlorophyll content in rice leaves, this study focuses on rice cultivars, and employs spectral technology as the foundational research tool. The study utilizes machine learning methodologies and information extraction techniques to identify characteristic bands and parameters for chlorophyll content in rice leaves. We have compared various analytical algorithms and investigated the predictive efficacy of hyperspectral and fluorescence spectral imaging methods to determine chlorophyll content in rice leaves. A fused CNN+LSTM model was employed, which accurately predicts the chlorophyll content in rice leaves. This research contributes to the non-destructive automatic monitoring and rational management of plant growth, thereby promoting the advancement of precision agriculture.

## 2. Plant Materials and Methods

### 2.1. Measurement of Hypespectral and Fluorescence Data

Figure S1 depicts the experimental region, which is located on a farm in the Yucheng District, Ya’an City, Sichuan Province (29.9890 N, 102.9820 E). The type of rice we selected was Ya5You5217, which is a rice variety selected and bred by the College of Agriculture of Sichuan Agricultural University. The climatic type is that of the western boundary of the Sichuan Basin—humid subtropical monsoon. The annual average temperature ranges from 14.1°C to 17.9 °C, and rainfall is abundant, with most places receiving more than 1000 mm. There are 10 sampling fields in the test area, with a single sampling field size of 12 × 8 m.

The GaiaSorter Hyperspectral Sorter is used to capture hyperspectral image data from rice in situ. Its key components are a unified tungsten-bromine light source, a spectral camera, and an electronically controlled mobile platform with a spectral band range of 387 nm to 1034 nm. Gaia series and aFluo series fluorescence spectral detection systems have been used to acquire fluorescence spectral image data of rice. The core components include a Gaiafluo VN-HR spectral camera, xenon light source, and fluorescence filter. Spectral data were measured directly with two sets of four LSTS-200 tungsten bromine lamp uniform light sources. A white reference plate with 99% reflectance was used for calibration before and after measurements. The spectral reflectance of three parts of rice leaves, namely, leaf tip, leaf center, and leaf occiput, were measured and their average values were used as the spectral reflectance of the samples. The sampling interval was 0.1 s and the spectrometer was calibrated every 15 min with a white reference plate.

The working principle is to irradiate the object to be measured (sample) placed on the electronically controlled moving platform (or conveyor belt) through the light source, the reflected light of the sample is captured by the spectral camera through the lens, and a one-dimensional image as well as spectral information is obtained, and with the electronically controlled moving platform (or conveyor belt) to drive the sample to run continuously, so as to obtain continuous one-dimensional image as well as the real-time spectral information. For both hyperspectral and fluorescence spectra measurements, the diffuse light source was placed directly above the measured sample at the same distance from the sample. The spatial uniformity of illumination was greater than or equal to 90%.

**Figure 1:**
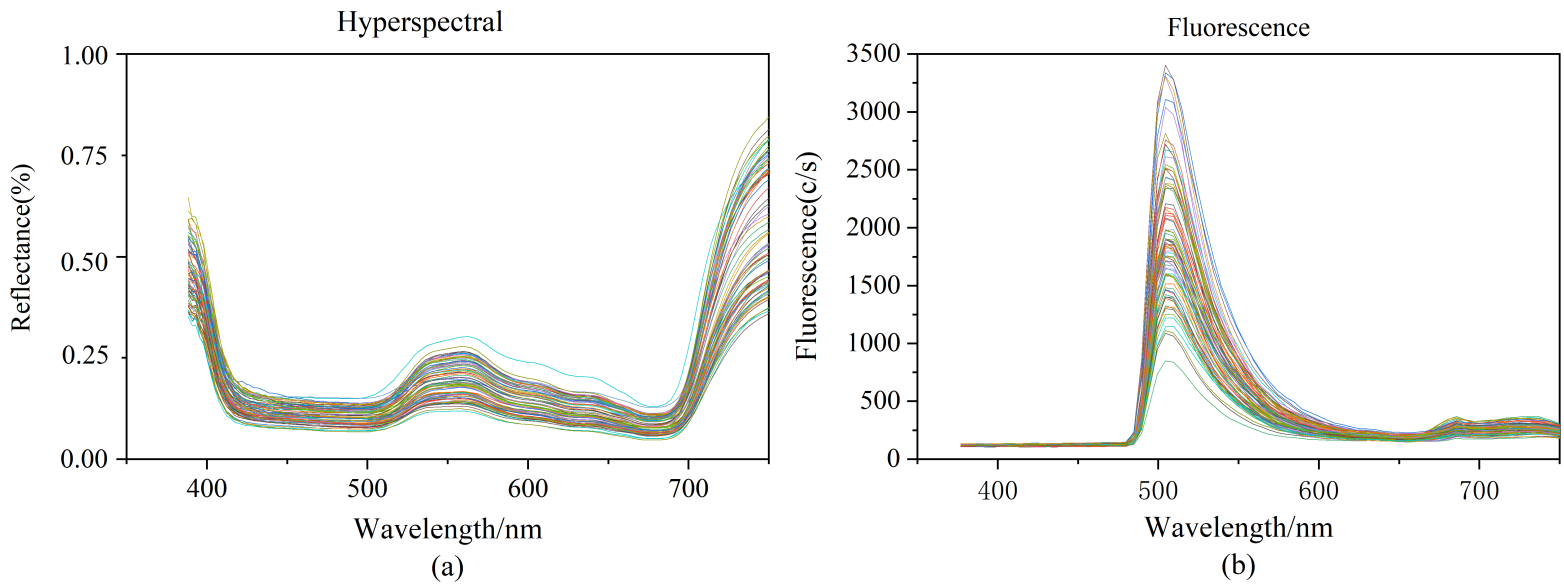
(**a**): Raw hyperspectral image data; (**b**): Raw fluorescence spectral image data.

We cut normal leaves from rice plants during normal growth. Because of the differences in chlorophyll content in the tip, occiput, and middle of the leaf of rice leaves, we sampled these three positions separately to ensure the reasonableness of our data. Three areas of interest (ROI) were chosen for each rice leaf based on the tip of the leaf, the middle of the leaf, and the back of the leaf. Each ROI was 15 × 15 pixels in size, and the raw spectral values of the samples were calculated by averaging the ROI values of the three portions of each sample. Figure 1a depicts the hyperspectral curves of the 90 rice samples. The hyperspectral has 256 spectral bands ranging in wavelength from 387.15 nm to 1034.99 nm. According to Figure 1a chlorophyll and other pigments in rice leaves absorb weakly near the red part of the spectrum, creating absorption valleys at 500 and 670 nm. The reflection peaking at 550 nm are mostly caused by the rice leaves’ high absorption of green light.

The fluorescence spectrums contained 125 spectral bands ranging in wavelength from 376.80 nm to 1011.05 nm. This wavelength range is green light (wavelength 500–560 nm), which is mainly absorbed by chlorophyll. The studies were carried out with a 495 nm fluorescence filter, and after passing through the excitation filter at 390 nm, the resulting fluorescence spectrum pictures revealed obvious wave peaking at about 510 nm, as well as inconspicuous wave peaking at around 690 nm and 740 nm. Varied samples contain varied chlorophyll contents in the hyperspectral and fluorescence spectra, resulting in variances in the spectral curves.

Before building a model with machine learning algorithms, the data was frequently separated into samples, which helps to increase the predicted accuracy of the model while also maintaining the model’s stability [29]. The Kennard Stone model was employed in this investigation [30,31]. The Kennard Stone algorithm first chooses the two samples with the greatest Euclidean distances from the data to be included in the training set, then computed the Euclidean distances between the remaining data and the chose data, and finally included the two samples with the greatest distances from the known samples in the training set. The above calculation is repeated to select representative samples until the required number of training samples is met, which helps to improve the model’s computational efficiency and generalizability. Table 1 shows the specific information for the 90 samples, which are separated into the training set and the test set in a 2:1 ratio.

Table 1 shows that the training set’s chlorophyll content ranges from 32.4 μg/cm^2^ to 47.8 μg/cm^2^ while the test set’s chlorophyll content ranges from 33.6 μg/cm^2^ to 48.2 μg/cm^2^. The test set’s standard deviation, 1.62 μg/cm^2^, is lower than the training set’s, 1.95 μg/cm^2^, indicating that the data distribution in the test set is more concentrated.

### 2.2. Invasive Measurement of Chlorophylls

Three sections of each rice leaf sample were taken from top to bottom in the experimental plot for the measurement of chlorophyll content in the rice samples; each section was cut into pieces of less than 1 cm, and samples weighing 2 g were selected and placed in triangular vials. As diffusate, 95% ethanol was poured into a 100 mL volumetric flask and filled to capacity. A total of 5 mL of the solution was pipetted into a 50 mL volumetric flask and fixed at 50 mL with 95% ethanol after vigorous shaking. Once the chlorophyll had been completely extracted, its colorimetric content was evaluated using a spectrophotometer. The chlorophyll content of the three regions of each sample leaf was averaged to determine the average chlorophyll content of the rice leaf [32].

### 2.3. Experimental Techniques and Protocols

Savitzky–Golay convolutional smoothing (SG) [33], a common preprocessing approach for hyperspectral data, was used to reduce noise and interferences of image acquisition environment. Figure S2 illustrates the data before and after preprocessing.

Mean-variance normalization (Z-score normalization) is a commonly used data processing method in machine learning [34]. Data normalization is a common machine learning method. The values of different bands of different samples have obvious differences, and using data normalization cannot change the data distribution while limiting the data to a small range, as depicted in Figure S3. Normalizing the spectral data of the samples facilitates the convergence of the model. The scaled data size helps to reduce the running memory during model training and improve the training efficiency. The normalized data helps the model find a better solution.

### 2.4. Feature Extraction Methods

Preprocessing only changes the data, but not the dimension of the data, and the processed data still has high dimensionality and redundant variables. Different bands show different correlations for chlorophyll content, so it is necessary to use feature extraction algorithms to filter out the feature variables with higher correlations and eliminate irrelevant variables. In this study, the bootstrapping soft threshold method bootstrapping soft shrinkage (Boss) was chosen overall [35], which is used to filter out noise, highlight key features, and improve the resolution of the data. A total of six feature extraction algorithms, competitive adapative reweighted sampling (CARS), Iteratively Variable Subset Optimization (IVSO), Model Adaptive Space Shrinkage (MASS), and Interval Variable Iterative Space Shrinkage Approach (IVISSA) were selected to process the spectral data and increase the prediction accuracy [36].

### 2.5. Modeling

#### 2.5.1. Convolutional Neural Networks and Long Short-Term Memory

**Figure 2:**
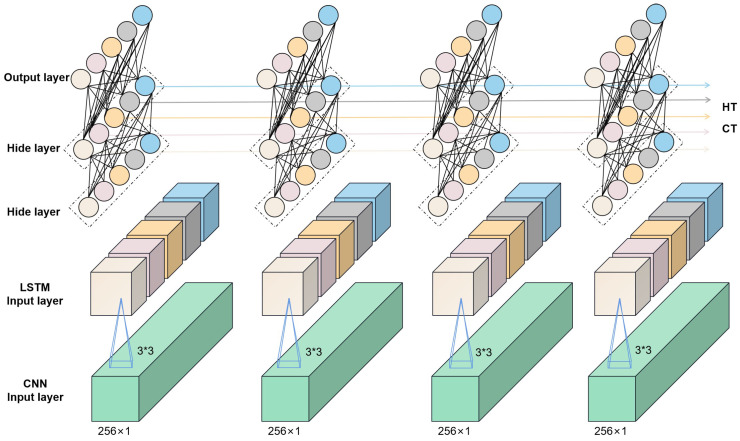
Schematic representation of the CNN+LSTM network structure.

Convolutional neural networks (CNNs) are multilayer perceptrons for simulating neurons that can be deepened by continuously deepening the layers of the perceptron, which is why they are also referred to as deep learning [37]. Convolutional neural networks are powerful and can be used for image classification [38], text analysis [39], microplastics in soil [40], and other areas. A complete convolutional neural network consists of an input layer, an output layer, a convolutional layer, and a fully connected layer. Generally, the input layer receives the preprocessed data and passes them to the subsequent convolutional layer for feature extraction. The convolutional layer is the central functional layer of the convolutional neural network, which contains multiple convolutional kernels for feature extraction. The pooling layer is a special type of convolutional layer that usually pools the data after each convolutional operation. The pooling layer can play a role in compressing the parameters and data, which helps to improve the accuracy of the model and increase the efficiency of the model operation. Long short-term memory (LSTM) is a network specialized in sequential data processing and used in stock price prediction [41], text comprehension and other prediction tasks. The use of LSTM networks for accurate spectral predictions also has good potential as a result of the fact that spectral data are also continuous between 400 and 1000 nm. Convolutional neural networks have higher feature extraction capabilities; however, they are generally independent in computation and can only extract features for a section of the data, frequently neglecting the correlation before and after the data. In this study, a fusion network comprising CNN and LSTM is utilized to assure the effectiveness of feature extraction while boosting the model’s ability to capture the information before and after the data, which helps increase the accuracy of model’s prediction. Figure 2 depicts the network. Furthermore, this study conducts a comparative analysis of three prevalent machine learning algorithms—linear regression, decision tree regression, and XGBoost (eXtreme Gradient Boosting)—to elucidate the superiority of the proposed method in assessing the chlorophyll content within rice leaves ([42,43,44]).

#### 2.5.2. Model Analysis

When there is a significant linear relationship in the data, the predictive accuracy of the model constructed using the linear regression method is very accurate. The measured hyperspectral/fluorescecne spectral features contain a large number of nonlinear relationships, which is the reason why numerous experiments in linear regression perform generally. In contrast, the regression tree can learn non-linear relationships and is also highly robust to outliers. Therefore, the regression tree model basically outperforms the linear regression model in all experimental metrics. And XGBoost adopts the idea of integrated learning, which effectively avoids the overfitting problem that occurs in the regression tree model. Therefore, its performance is optimal among these three comparative methods. For the CNN+LSTM fusion network method proposed in this paper, it achieves very impressive performance with the same input features as the above comparison experiments. Meanwhile, drawing on previous ideas, we have conducted secondary feature extraction experiments on the combination of CNN+LSTM+IVSO. Since CNN itself has certain feature extraction ability, more information can be extracted for prediction when there are more feature bands. Therefore, we obtain the optimal experimental combination: CNN+LSTM+IVSO−IVISSA.

### 2.6. Evaluation Indicators

To analyze the classification outcomes in this work, the training set root mean square error (RMSE-Train), test set root mean square error RMSE-Test), and relative analysis error RPD are utilized as assessment metrics ([45,46,47,48]). The formula for the metrics used is as follows:

(1) $$\mathrm{RMSE}=\sqrt{\frac{\sum_{i=1}^{n}{\left(f_{t}-y_{t}\right)}^{2}}{n}}$$

(2) $$\mathrm{RPD}=\frac{Std_{p}}{RMSEP}$$

(3) $$Std_{p}=\sqrt{\frac{1}{n-1}\sum_{i=1}^{n}{\left(y_{t}-\bar{y}\right)}^{2}}$$

The square root of the ratio between the square of the predicted value’s divergence from the true value and the total number of samples N is RMSE. In Equation (1), $f_{t}$ is the predicted value of the sample and $y_{t}$ is the actual value of the sample determined by the standard method. In Equation (2), $Std_{p}$ is the standard deviation. The formula is as in (3), where $\bar{y}$ is the average of the actual measured value of the sample. The lower the RMSE score is, the greater the model’s prediction accuracy will be. RPD is the ratio of the data standard deviation to the root mean square error.4.2 Effectiveness of feature extraction.

## 3. Results

### 3.1. Feature Extraction Results

**Figure 3:**
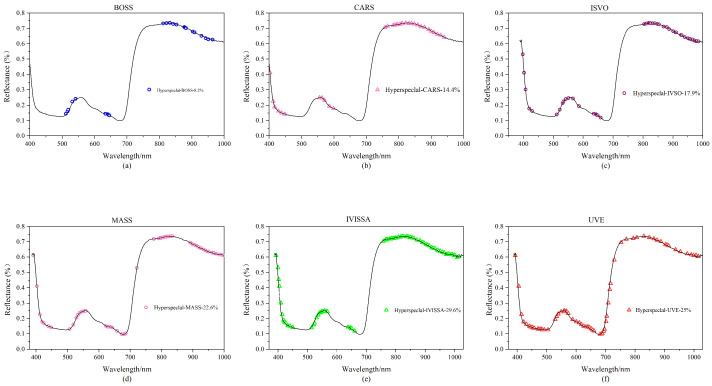
Hyperspectral feature band map created using various feature extraction algorithms: (**a**) BOSS; (**b**) CARS; (**c**) IVSO; (**d**) MASS; (**e**) IVISSA; and (**f**) UVE.

In this study, 90 rice leaf samples were employed, each of which had spectral information from 256 different bands, and a substantial quantity of redundant information is always present in sample data at high latitude. Six feature extraction techniques (BOSS, CARS, IVSO, MASS, IVISSA, UVE) are utilized for feature extraction of hyperspectral data to increase operating efficiency and prediction accuracy. The approach with the best results is chosen for the next trial based on the experimental findings, and the results are shown in Figure 3.

As shown in Figure 3a, 21 feature variables are generated following feature extraction of the spectrum data with the BOSS algorithm, accounting for 8.2% of the total number of hyperspectral variables. The retrieved feature bands are primarily in the 500–650 nm and 800–1000 nm ranges. The feature variables are concentrated near the wave crests and troughs, but their distribution is insufficiently uniform. As shown in Figure 3b, after applying the CARS algorithm, a total of 37 feature bands are created, accounting for 14.4% of the total hyperspectral variables. The feature bands are predominantly concentrated between 400–600 nm and 750–900 nm, and the frequency of the feature bands outside the wave crest is higher. As shown in Figure 3c, the IVSO algorithm recovers 46 feature bands for the original data, accounting for 17.9% of the total number of hyperspectral variables. The feature distribution is quite uniform, with the majority of features gathered between 800 and 1000 nm. The MASS algorithm selects 58 feature bands from the original data, accounting for 22.6% of the total hyperspectral variables, as shown in Figure 3d. As illustrated in Figure 3e, the IVISSA method yields 76 feature bands, accounting for 29.6% of the total number of hyperspectral variables. Among all feature extraction techniques, it obtains the most feature bands, and the feature bands are uniformly distributed. The EIS method generates 64 feature bands, accounting for 25% of the total number of hyperspectral variables; the feature band distribution is illustrated in Figure 3f.

The results of six feature extraction techniques used to extract features from fluorescence spectrum data are displayed in Figure S4. As illustrated in Figure S4a, when BOSS feature extraction is performed on the original fluorescence spectral data, the minimum number of feature bands obtained is only 11, accounting for 8.8% of the total number of feature bands in the fluorescence spectra. The main feature bands are clustered near the 510 and 700 nm peaks. Figure S4b depicts the results of CARS feature extraction from the original fluorescence spectrum data. A total of 14 feature bands were discovered, accounting for 11.2% of all feature bands in the fluorescence spectrum.The majority of the feature bands are approximately 70 nm in wavelength. As shown in Figure S4c, the IVSO algorithm discovers 19 feature bands from the original fluorescence spectral data, accounting for 15.2% of the total number of fluorescence spectral features. The feature bands are mostly spread uniformly near the wave crests and troughs. The MASS algorithm extracted a total of 24 feature bands for the original data, accounting for 19.2% of the total number of hyperspectral variables, as shown in Figure S4d, with the main feature bands focused at the wave crests at 510 nm and 700 nm.The IVISSA feature extraction of the original fluorescence spectral data produced the most feature bands, with a total of 38 feature bands accounting for 30.4% of the total number of fluorescence spectral features, and the feature band range is between 475–600 nm and 700–800 nm, as shown in Figure S4e. Following the application of the EIS method, a total of 27 feature bands are retrieved, accounting for 21.6% of the total number of feature bands in the fluorescence spectrum, as shown in Figure S4f.

### 3.2. Validity of the Modeling Methods

#### 3.2.1. Linear Regression

The six types of hyperspectral feature variables collected above were separately entered into the linear regression model, and the prediction results obtained are displayed in Table 2.

As shown in Table 2, the linear regression models built with the six types of feature spectra are generally less effective. The IVISSA algorithm has the highest number of features after extraction, but the RMSE train indicator is the highest at 0.82. The reason for this is that there are still some redundant data in the feature variables extracted by the IVISSA algorithm. The most effective feature extraction method is the IVSO algorithm with an RMSE train of 0.60 and an RPD of 2.04, which is also the highest value. The linear regression model, which can only capture the relationship between variables based on a linear relationship between data, has weak performance on data with a hierarchical structure. The results show that the linear regression method has a better predictive ability for high-dimensional and complex data. The results of prediction performance obtained by inputting each of the six types of fluorescence spectral features extracted above into the linear regression model are shown in Table 3. The IVSO algorithm, which performs better on hyperspectral data, performs poorly on fluorescence spectral data, and the RMSE train reaches the highest value of 0.74 among the comparison algorithms. From the RMSE train index, IVISSA is the algorithm with the best feature extraction effect, with the smallest RMSE train of 0.54. However, the corresponding RMSE train of the IVISSA algorithm is the highest at 0.83, indicating that the prediction model is overfitted and has poor generalization performance. In terms of RPD index, the best effect of feature extraction is the Boss algorithm, which achieves a maximum RPD of 2.11. The prediction effect of the fluorescence spectral data is better than that of the hyperspectral data.

#### 3.2.2. Regression Tree

The constructed regression tree model is better at capturing the nonlinear relationship in the data, and it is obviously lower than the above model in terms of training and prediction speed. However, the prediction accuracy is relatively low, and due to the complex structure of the tree model, more data is often required to determine the best parameters. The indices of the prediction results are shown in Table 4. The RMSE train index of the regression tree model is significantly lower than the RMSE test index, which proves that the regression tree model has a generalization ability and the possibility of overfitting. The CARS feature extraction algorithm performs optimally in the regression tree model, and both RMSE-Train and RMSE-Test reach the minimum, and the RPD value is the highest among the comparison models, which proves the effectiveness of the selected features. The IVISSA algorithm, on the other hand, performs poorly in this section of the experiment.

The results of the regression tree prediction model based on fluorescence spectral data are shown in Table 5. Overall, the indicators of each algorithm are better than the regression tree prediction model for hyperspectral data. The best feature extraction algorithm is IVSO with the lowest RMSE train metric of 0.60, and the RMSE test metric also performs well, with the largest RPD value of 2.12 among the algorithms, while the IVISSA algorithm performs poorly, with the highest values of both the RMSE train and RMSE test metrics and the lowest corresponding RPD value of 1.93. also has the smallest value of 1.93.

#### 3.2.3. XGBoost

The predictive performance of the six types of hyperspectral feature variables input to the XGBoost model is shown in Table 6. XGBoost uses the practice of random forests to sample different columns for training, which is able to avoid overfitting while reducing the training time. The XGBoost model has the lowest performance on spectral features extracted by the IVSO algorithm, and both the RMSE train and RMSE test are the lowest among the comparison methods. The CARS algorithm is the second most effective and the UVE feature extraction algorithm is the most ineffective.

The six types of fluorescence spectral variables were input into the XGBoost model, whose prediction results are shown in Table 7. For fluorescence spectral data, the IVSO algorithm again performs well, with RMSE-Train and RMSE-Test achieving the best results in all comparison experiments. While the IVISSA feature extraction algorithm did not perform well, the regression prediction model for fluorescence spectral data performed better than the regression prediction model for hyperspectral data.

#### 3.2.4. CNN+LSTM

In this work, when the fusion network based on convolutional neural network and LSTM is developed, a learning rate of 0.01 is selected, the relu function is used as the activation function, and the cross entropy loss function is used as the objective function of the model. The six types of spectral feature variables extracted as described previously are respectively input to the convolutional neural network, and the obtained prediction result indices are shown in Table 8.

As seen from Table 8, the lowest RMSE train of the convolutional neural network built with six types of spectral feature variables is 0.32, which corresponds to the IVISSA feature extraction method, while the RMSE train of the IVSO algorithm, which performs better in the previous model, is 0.36. The lowest value of the RMSE train is also found in the features extracted by the IVISSA algorithm, while the maximum RPD value is 2.2. The suggestion that this round of experiments seems to contradict the previous experiments is that the convolutional neural network also plays a role in feature extraction during the convolutional process. Again, feature extraction is more beneficial for samples with a larger number of features to obtain more valid information. For samples with a smaller number of features, re-feature extraction may eliminate the effective information, which in turn affects the results. To further investigate this hypothesis, a secondary feature extraction experiment is conducted.

Based on fluorescence spectral data, the results of the different prediction indices of the convolutional neural network constructed with six types of spectral features are shown in Table 9. When spectral data are used to construct the model, the results obtained are generally better than those obtained with the hyperspectral data. Consistent with the results of the hyperspectral data, the IVISSA feature extraction algorithm is the most effective, with the lowest RMSE train value of 0.30, the lowest RMSE test value of 0.34, and the highest corresponding RPD value of 2.56, while the Boss algorithm performs the worst.

In the conventional realm of hyperspectral prediction, it is posited that the amalgamation of features derived from the extraction of multifaceted attributes can enhance the veracity of the resultant predictions. This assertion is predicated on the premise that integrating diverse spectral characteristics can provide a more comprehensive representation of the data, thereby facilitating improved analytical outcomes [49]. In Table 10, the IVSO feature extraction algorithm was chosen, which performs well in most models, as the benchmark and perform feature extraction again on the data after performing feature extraction once and input it to the convolutional neural network for training. The results are shown in Table 6. The number of feature bands obtained after the second feature extraction is reduced. From Table 6, it can be seen that the feature extraction algorithm obtained by combining IVSO-IVISSA performs the best, with the number of feature bands at 37 and the maximum RPD value at 2.57. Compared with the other experiments, the effect of the feature bands obtained by the secondary feature extraction algorithm is much better than that of the feature extraction algorithm using only the primary feature extraction algorithm. Compared with the experiments in Table 9, although the RMSE values all decrease, the degree of change in the RMSE values of the IVSO-IVISSA method is less than that of the other centralized feature extraction algorithms, while the IVSO-IVISSA method obtains more bands. This shows that the CNN+LSTM network is still superior, but when the number of features decreases, it has a certain negative impact on the performance of the network.

**Figure 4:**
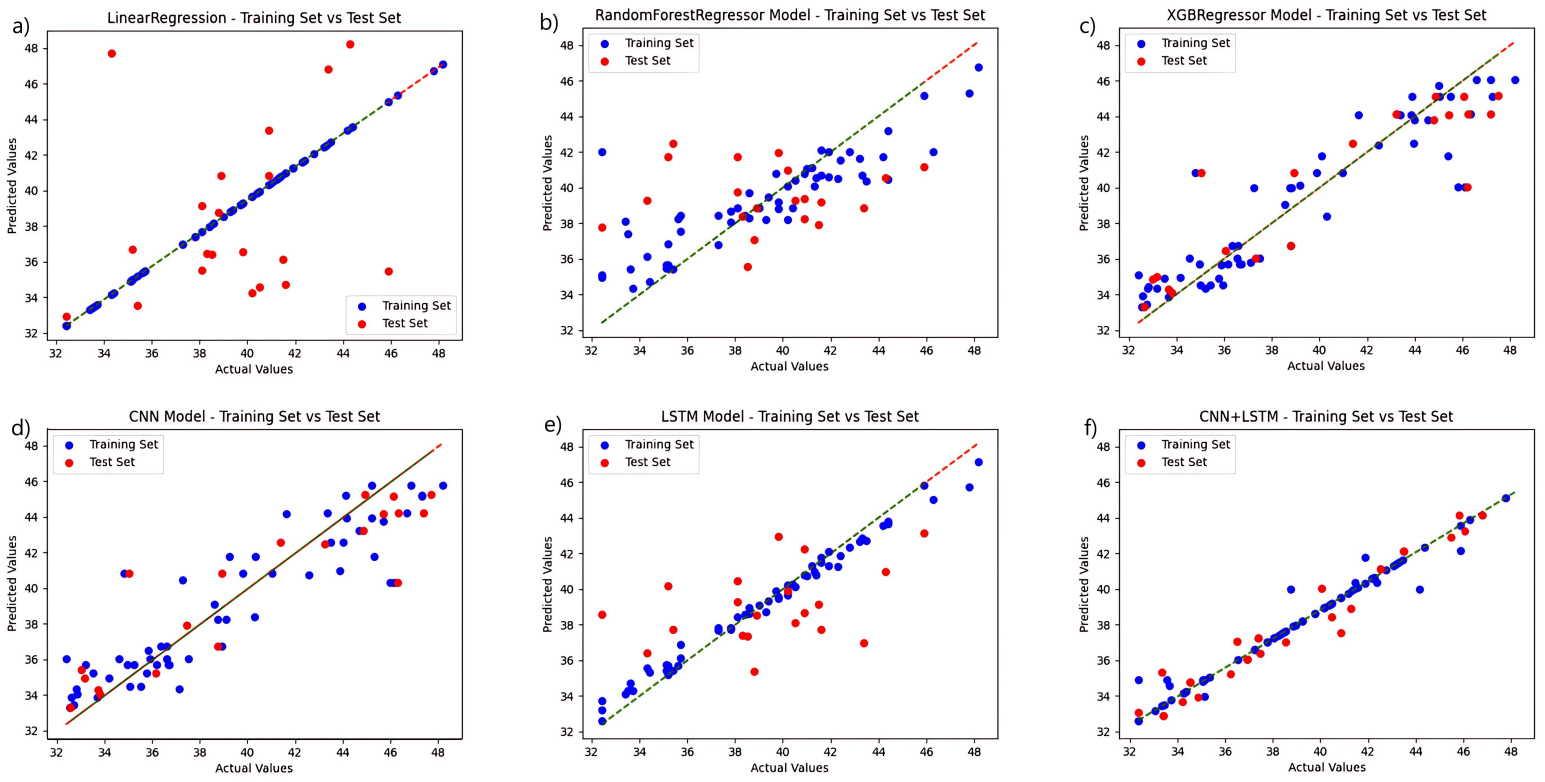
Comparison of the prediction functions of six models in the same hyperspectral feature band: (**a**) LinearRegression; (**b**) RandomForestRegressor; (**c**) XGBRegressor; (**d**) CNN; (**e**) LSTM; and (**f**) CNN+LSTM.

To validate the performance of the fusion network chosen for this study, a regression analysis were conducted independently for each model based on the selection of the ideal feature extraction algorithm, and the chlorophyll prediction results are displayed in Figure 4. The CNN+LSTM fusion technique, among various techniques, outperforms the other networks in terms of prediction accuracy. The linear regression algorithm and the LSTM-only algorithm outperform the other three networks in the training set, but the linear regression approach performs poorly in the test set, while the LSTM prediction set outperforms them. The CNN algorithm performs relatively well in the training and prediction results of test set. The preceding experiments demonstrate the fusion network’s advantage.

**Figure 5:**
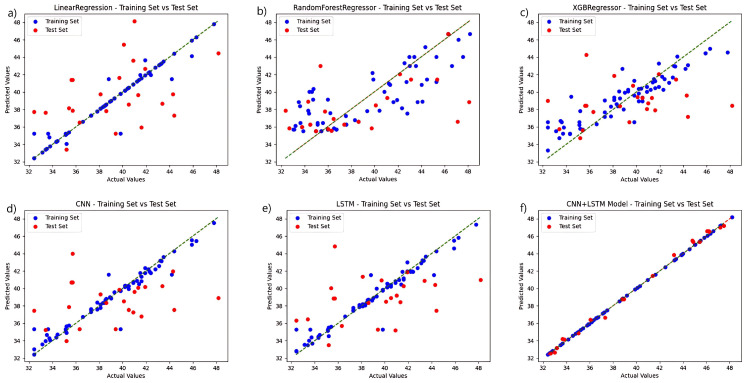
Comparison of the prediction functions of six models in the same fluorescence spectral feature band: (**a**) LinearRegression; (**b**) RandomForestRegressor; (**c**) XGBRegressor; (**d**) CNN; (**e**) LSTM; and (**f**) CNN+LSTM.

Regression analysis was carried out separately for each model using fluorescence spectral data, and the results are displayed in Figure 5. In a direct comparison, the CNN+LSTM fusion algorithm surpasses the control group in both the training and test sets. The model that utilizes solely CNN and LSTM performs better than the regression tree and XGBoost algorithms in the training set and maintains better overall performance in the test set. The regression tree approach, coupled with the XGBoost training process, performed poorly, failing to effectively learn the probable relationships within the data. When the models are developed with fluorescence spectrum data, the six regression prediction models exhibit a slight improvement over those developed with hyperspectral data.

Given the limited experimental data available, we used five-fold cross-validation within the established context of fluorescence spectral data and the dual feature extraction methods of IVSO-IVISSA to compare the performance of the models discussed in this paper. The results, as depicted in Table 11, confirm the superior performance of the methodologies proposed in this study.

## 4. Discussion

This study collects hyperspectral and fluorescence spectral data from rice leaves to examine the spectral features of chlorophyll. Six feature extraction algorithms, including regression trees, linear regression, and the XGBoost model, are employed to assess the nitrogen content in rice leaves. The gathered data are normalized, and convolution smoothing is applied to reduce noisy samples. The findings suggest that fluorescence spectroscopy is superior in detecting chlorophyll concentration in rice leaves. Utilizing the strengths of both CNN and LSTM for learning non-linear and multidimensional data, the CNN+LSTM fusion model presented in this paper outperforms other machine learning models in accurately predicting chlorophyll content. This study also verifies the effectiveness of secondary feature extraction and identifies the optimal combination for chlorophyll prediction in rice leaves: the CNN+LSTM+IVSO−IVISSA method based on fluorescence spectral data. A comparison with other studies on chlorophyll prediction using machine learning methods—most of which involve standard approaches such as XGBoost, SVM, and Random Forest—demonstrates the capability of machine learning to effectively predict chlorophyll content. Beyond rice, the task of chlorophyll prediction is relevant to fields such as water quality, corn, tea, and more, as shown in Table 12. The paper underscores that spectral imaging technology combined with machine learning can facilitate rapid, accurate, and nondestructive detection of chlorophyll in rice leaves, offering a simpler and time-saving measurement method. Limited by equipment constraints, it is challenging to acquire leaf spectral band information in field conditions during practical applications. Consequently, designing portable spectral sensors is an issue that urgently needs to be addressed. Future research will expand the types of samples, including the measurement of rice canopies. Moreover, while this study focuses on a single developmental stage of rice, future work will develop multiple growth monitoring models for different developmental stages to elucidate the rice growth process. The current study, based on field experiments, examines the physiological and biochemical parameters of rice at the leaf level using hyperspectral diagnostics. Its practical application awaits further validation. Due to the commonality of spectral mechanisms, the findings will be verified using unmanned aerial vehicles (UAVs) and satellite remote sensing data to ensure the broader applicability of the study’s conclusions.

## 5. Conclusions

This study collected hyperspectral and fluorescence spectral data of rice leaves and analyzed the spectral characteristics of chlorophyll in rice leaves. Six feature extraction algorithms, utilizing regression trees, linear regression, XGBoost models, and other machine learning models, were employed to detect the chlorophyll content in rice leaves. The collected data were normalized, and convolutional smoothing was applied to reduce noise samples. The results indicate that fluorescence spectra have advantages in detecting chlorophyll content in rice leaves. In comparison to existing machine learning models, the CNN+LSTM fusion model used in this study more accurately predicts the chlorophyll content in rice leaves. The CNN+LSTM fusion model, applicable to fluorescence spectral data, exhibited optimal RMSE-Train, RMSE-Test, and RPD indicators, with values of 0.26, 0.29, and 2.64, respectively. This demonstrates that the combination of spectral imaging technology and machine learning regression prediction methods enables the rapid and accurate non-destructive detection of chlorophyll in rice leaves, providing a simpler and time-saving method for measuring chlorophyll in rice leaves. The detection method devised in this study relies on specific spectral equipment, and obtaining real-time spectral band information from rice leaves in the field presents certain challenges. In future work, we will further diversify sample types, considering chlorophyll content measurements in both rice leaves and the rice canopy. Additionally, this study focused solely on a specific developmental stage of rice; subsequent efforts will establish growth monitoring models for different stages of rice development to reveal the growth mechanisms of rice. This research, conducted based on field experiments, specifically addressed leaf-scale physiological and biochemical parameters of rice using hyperspectral diagnosis. The design of portable spectral sensors is advantageous for the real-time acquisition of leaf spectral band information in the field and constitutes an essential step toward the future goal of achieving efficient and rapid chlorophyll detection in rice leaves. The practical application awaits verification, and given the spectral mechanism’s commonality, future efforts will involve unmanned aerial vehicles and satellite remote sensing data to validate the research results, aiming to ensure the broader applicability of the conclusions drawn from the study.

## Figures and Tables

**Table 1:** Summary of hyperspectral and fluorescence spectroscopy data.

	Record	Sample Number	Minimum Value (μg/cm^2^)	Maximum Values (μg/cm^2^)	Average Value (μg/cm^2^)	(Statistics) Standard Deviation (μg/cm^2^)
hyperspectral	Training set	60	32.4	47.8	38.9	1.95
	Test set	30	33.6	48.2	39.1	1.62
Fluorescence spectroscopy	Training set	60	32.4	47.8	38.9	1.95
	Test set	30	33.6	48.2	39.1	1.62

**Table 2:** Linear regression prediction results based on hyperspectral data.

Feature Extraction Method	The Number of Characteristic Variables	RMSE Train	RMSE Test	RPD
Chief	21	0.71	0.85	1.76
CARS	37	0.65	0.79	1.97
IVSO	46	0.60	0.71	2.04
IVISSA	76	0.82	0.97	1.84
MASSES	58	0.79	0.82	1.93
EIS	64	0.73	0.89	1.75

**Table 3:** Linear regression prediction results based on fluorescence spectral data.

Feature Extraction Method	The Number of Characteristic Variables	RMSE Train	RMSE Test	RPD
Chief	11	0.62	0.69	2.11
CARS	14	0.61	0.71	1.87
IVSO	19	0.74	0.80	1.37
IVISSA	38	0.54	0.83	1.61
MASSES	24	0.66	0.76	1.53
EIS	27	0.68	0.77	1.75

**Table 4:** Regression tree regression prediction results based on hyperspectral data.

Feature Extraction Method	The Number of Characteristic Variables	RMSE Train	RMSE Test	RPD
Chief	21	0.68	0.73	1.83
CARS	37	0.63	0.70	2.03
IVSO	46	0.65	0.73	1.88
IVISSA	76	0.77	0.86	1.95
MASSES	58	0.71	0.81	2.1
EIS	64	0.67	0.76	1.90

**Table 5:** Regression tree prediction results based on fluorescence spectral data.

Feature Extraction Method	The Number of Characteristic Variables	RMSE Train	RMSE Test	RPD
Chief	11	0.65	0.70	2.06
CARS	14	0.64	0.67	2.02
IVSO	19	0.60	0.68	2.12
IVISSA	38	0.72	0.76	1.97
MASSES	24	0.68	0.74	1.93
EIS	27	0.64	0.71	2.04

**Table 6:** XGBoost regression prediction results based on hyperspectral data.

Feature Extraction Method	The Number of Characteristic Variables	RMSE Train	RMSE Test	RPD
Chief	21	0.60	0.68	2.07
CARS	37	0.56	0.67	2.25
IVSO	46	0.54	0.63	2.27
IVISSA	76	0.57	0.69	2.17
MASSES	58	0.60	0.67	2.00
EIS	64	0.67	0.73	1.78

**Table 7:** XGBoost regression prediction results based on fluorescence spectral data.

Feature Extraction Method	The Number of Characteristic Variables	RMSE Train	RMSE Test	RPD
Chief	11	0.57	0.63	2.11
CARS	14	0.53	0.59	2.28
IVSO	19	0.50	0.56	2.34
IVISSA	38	0.52	0.60	2.08
MASSES	24	0.55	0.62	2.03
EIS	27	0.61	0.69	1.98

**Table 8:** CNN+LSTM prediction results based on hyperspectral data.

Feature Extraction Method	The Number of Characteristic Variables	RMSE Train	RMSE Test	RPD
Chief	21	0.47	0.52	2.06
CARS	37	0.41	0.46	2.23
IVSO	46	0.36	0.40	2.42
IVISSA	76	0.32	0.38	2.53
MASSES	58	0.44	0.47	2.19
EIS	64	0.37	0.43	2.13

**Table 9:** CNN+LSTM prediction results based on fluorescence spectral data.

Feature Extraction Method	The Number of Characteristic Variables	RMSE Train	RMSE Test	RPD
Chief	11	0.46	0.53	2.09
CARS	14	0.40	0.47	2.25
IVSO	19	0.33	0.38	2.47
IVISSA	38	0.30	0.34	2.56
MASSES	24	0.38	0.41	2.27
EIS	27	0.34	0.39	2.18

**Table 10:** CNN+LSTM and quadratic feature extraction prediction results based on fluorescence spectral data.

Feature Extraction Method	The Number of Characteristic Variables	RMSE Train	RMSE Test	RPD
IVSO Boss	19	0.39	0.43	1.96
IVSO-CARS	22	0.37	0.40	2.09
IVSO-IVISSA	37	0.26	0.29	2.64
IVSO-MASS	27	0.32	0.38	2.13
IVSO-UVE	32	0.29	0.34	2.28

**Table 11:** Model performance comparison.

Data Categories	Cross- Validation	Average RMSE-Train	Average RMSE-Test
Linear Regression	✕	0.18	0.74
Linear Regression	✓	0.16	0.87
Random Forest	✕	0.47	0.52
Random Forest	✓	0.47	0.51
XGBoost	✕	0.33	0.36
XGBoost	✓	0.34	0.38
LSTM+CNN	✕	0.26	0.29
LSTM+CNN	✓	0.25	0.27

**Table 12:** The comparison of the work of this paper with the work of other researchers.

Literature	Method	RMSE	RPD	Data
Shin, Y. [50]	XGBoost	3.93	-	Chlorophyll-a concentrations in the Nakdong River
	LSTM	4.69	-	
	Random Forest	3.12	-	
Sonobe, R. [51]	SVM	-	0.81	Tea leaf chlorophyll
	Random Forest	-	1.12	
De Amorim, F.D.L. [52]	Random Forest	0.35	-	Chlorophyll-a concentration
Narmilan, A. [53]	XGBoost	0.14	-	Canopy chlorophyll content in sugarcane crops
Tang, X.D. [54]	SVM	10.07	-	Chlorophyll A concentration inDonghu Lake
Our study	CNN+LSTM	0.26	2.64	Rice chlorophyll

## Data Availability

The data presented in this study are available on request from the corresponding author.

## Supplementary Materials

The following supporting information can be downloaded at: https://www.mdpi.com/article/10.3390/plants13091270/s1, Figure S1: The location of the research area; Figure S2: Comparison plot of data after SG convolution smoothing: (a) original hyperspectraldata, (b) hyperspectral data after convolution smoothing, (c) originalfluorescence spectral data, (d)fluorescence spectral data after convolution smoothing; Figure S3: Schematic representation of the data normalization process; Figure S4: The illustration of the characteristic bands of the fluorescence spectrum obtained by different feature extraction algorithms. (a): Feature Extraction Results of BOSS Method; (b): Feature Extraction Results of CARS Method; (c): Feature Extraction Results of IVSO Method; (d): Feature Extraction Results of MASS Method; (e): Feature Extraction Results of IVISSA Method; (f): Feature Extraction Results of UVE Method.

## References

[1] Romero F.M., Gatica-Arias A. (2019). CRISPR/Cas9: Development and Application in Rice Breeding. Rice Sci..

[2] Hefferon K.L. (2015). Nutritionally Enhanced Food Crops; Progress and Perspectives. Int. J. Mol. Sci..

[3] Khalid H.M., Mahmod I.F., Barakbah S.S., Osman N. (2018). Impact of Water Management on Fertilizer and Tillering Dynamics in Rice. Int. J. Agric. Biol..

[4] Ban S., Liu W., Tian M., Wang Q., Yuan T., Chang Q., Li L. (2022). Rice Leaf Chlorophyll Content Estimation Using UAV-Based Spectral Images in Different Regions. Agronomy.

[5] Molina L., Lapis J.R., Sreenivasulu N., Cuevas R.P.O. (2019). Determination of Cadmium Concentration in Milled and Brown Rice Grains Using Graphite Furnace Atomic Absorption Spectrometry. Methods Mol. Biol..

[6] Krasnovskii A.A., Bystrova M.I. (1960). Studies on chlorophyll synthesis in homogenates of etiolated leaves by means of a fluorescence spectrophotometric method. Biokhimiia.

[7] Tan L.H., Zhou L., Zhao N., He Y., Qiu Z.J. (2021). Development of a low-cost portable device for pixel-wise leaf SPAD estimation and blade-level SPAD distribution visualization using color sensing. Comput. Electron. Agric..

[8] Lee S., Masclaux-Daubresse C. (2021). Current Understanding of Leaf Senescence in Rice. Int. J. Mol. Sci..

[9] Kandpal K.C., Kumar A. (2023). Migrating from Invasive to Noninvasive Techniques for Enhanced Leaf Chlorophyll Content Estimations Efficiency. Crit. Rev. Anal. Chem..

[10] Zhao J.W., Wang K.L., Ouyang Q., Chen Q.S. (2011). Measurement of Chlorophyll Content and Distribution in Tea Plant’s Leaf Using Hyperspectral Imaging Technique. Spectrosc. Spectr. Anal..

[11] Jang S.H., Hwang Y.K., Lee H.J., Lee J.S., Kim Y.H. (2018). Selecting Significant Wavelengths to Predict Chlorophyll Content of Grafted Cucumber Seedlings Using Hyperspectral Images. Korean J. Remote. Sens..

[12] Cao Y.L., Jiang K.L., Wu J.X., Yu F.H., Du W., Xu T.Y. (2020). Inversion modeling of japonica rice canopy chlorophyll content with UAV hyperspectral remote sensing. Plos ONE.

[13] Feng H., Chen G.X., Xiong L.Z., Liu Q., Yang W.N. (2017). Accurate Digitization of the Chlorophyll Distribution of Individual Rice Leaves Using Hyperspectral Imaging and an Integrated Image Analysis Pipeline. Front. Plant Sci..

[14] Gitelson A.A., Merzlyak M.N. (1996). Signature Analysis of Leaf Reflectance Spectra: Algorithm Development for Remote Sensing of Chlorophyll. J. Plant Physiol..

[15] Liu H.H., Lei X.Q., Liang H., Wang X. (2023). Multi-Model Rice Canopy Chlorophyll Content Inversion Based on UAV Hyperspectral Images. Sustainability.

[16] Yang Y., Nan R., Mi T., Song Y., Shi F., Liu X., Wang Y., Sun F., Xi Y., Zhang C. (2023). Rapid and Nondestructive Evaluation of Wheat Chlorophyll under Drought Stress Using Hyperspectral Imaging. Int. J. Mol. Sci..

[17] Ruszczak B., Wijata A.M., Nalepa J. (2022). Unbiasing the Estimation of Chlorophyll from Hyperspectral Images: A Benchmark Dataset, Validation Procedure and Baseline Results. Remote. Sens..

[18] Baker N.R. (2008). Chlorophyll Fluorescence: A Probe of Photosynthesis In Vivo. Annu. Rev. Plant Biol..

[19] Malenovsky Z., Mishra K.B., Zemek F., Rascher U., Nedbal L. (2009). Scientific and technical challenges in remote sensing of plant canopy reflectance and fluorescence. J. Exp. Bot..

[20] Mishra A., Matous K., Mishra K.B., Nedbal L. (2009). Towards Discrimination of Plant Species by Machine Vision: Advanced Statistical Analysis of Chlorophyll Fluorescence Transients. J. Fluoresc..

[21] Mattila H., Valli P., Pahikkala T., Teuhola J., Nevalainen O.S., Tyystjärvi E. (2013). Comparison of chlorophyll fluorescence curves and texture analysis for automatic plant identification. Precis. Agric..

[22] Codrea M.C., Hakala-Yatkin M., Kårlund-Marttila A., Nedbal L., Aittokallio T., Nevalainen O.S., Tyystjärvi E. (2010). Mahalanobis distance screening of Arabidopsis mutants with chlorophyll fluorescence. Photosynth. Res..

[23] Tyystjärvi E., Nørremark M., Mattila H., Keränen M., Hakala-Yatkin M., Ottosen C.O., Rosenqvist E. (2011). Automatic identification of crop and weed species with chlorophyll fluorescence induction curves. Precis. Agric..

[24] Yang J., Shi S., Gong W., Du L., Zhu B., Ma Y.Y., Sun J. (2016). Laser induced fluorescence spectrum characteristics of paddy under nitrogen stress. Spectrosc. Spectr. Anal..

[25] Shen C., Feng Z., Zhou D. (2018). Analysing the effect of paddy rice variety on fluorescence characteristics for nitrogen application monitoring. R. Soc. Open Sci..

[26] Kang Z., Fan R., Zhan C., Wu Y., Lin Y., Li K., Qing R., Xu L. (2024). The Rapid Non-Destructive Differentiation of Different Varieties of Rice by Fluorescence Hyperspectral Technology Combined with Machine Learning. Molecules.

[27] Yang J., Sun J., Du L., Chen B., Zhang Z., Shi S., Gong W. (2017). Monitoring of Paddy Rice Varieties Based on the Combination of the Laser-Induced Fluorescence and Multivariate Analysis. Food Anal. Methods.

[28] Zhang H., Zhu L., Hu H., Zheng K., Jin Q. (2011). Monitoring Leaf Chlorophyll Fluorescence with Spectral Reflectance in Rice (*Oryza sativa* L.). Procedia Eng..

[29] Charilaou P., Battat R. (2022). Machine learning models and over-fitting considerations. World J. Gastroenterol..

[30] Wei X., He J., Zheng S., Ye D. (2020). Modeling for SSC and firmness detection of persimmon based on NIR hyperspectral imaging by sample partitioning and variables selection. Infrared Phys. Technol..

[31] Huang H., Hu X., Tian J., Jiang X., Luo H., Huang D. (2021). Rapid detection of the reducing sugar and amino acid nitrogen contents of Daqu based on hyperspectral imaging. J. Food Compos. Anal..

[32] Yang X., Lu X., Shi J., Li J., Ju W. (2022). Inversion of Rice Leaf Chlorophyll Content Based on Sentinel-2 Satellite Data. Spectrosc. Spectr. Anal..

[33] Schmid M., Rath D., Diebold U. (2022). Why and How Savitzky-Golay Filters Should Be Replaced. ACS Meas. Sci. Au.

[34] Tanaka T., Nambu I., Maruyama Y., Wada Y. (2022). Sliding-Window Normalization to Improve the Performance of Machine-Learning Models for Real-Time Motion Prediction Using Electromyography. Sensors.

[35] Zhang M., Guo J., Ma C., Qiu G., Ren J., Zeng F., Lu E. (2020). An Effective Prediction Approach for Moisture Content of Tea Leaves Based on Discrete Wavelet Transforms and Bootstrap Soft Shrinkage Algorithm. Appl. Sci..

[36] Beattie J.R., Esmonde-White F.W.L. (2021). Exploration of Principal Component Analysis: Deriving Principal Component Analysis Visually Using Spectra. Appl. Spectrosc..

[37] Ibrahim R., Shafiq M.O. (2023). Explainable Convolutional Neural Networks: A Taxonomy, Review, and Future Directions. ACM Comput. Surv..

[38] Huang S.Y., An W.J., Zhang D.S., Zhou N.R. (2023). Image classification and adversarial robustness analysis based on hybrid convolutional neural network. Opt. Commun..

[39] Lyu S.F., Liu J.Q. (2021). Convolutional Recurrent Neural Networks for Text Classification. J. Database Manag..

[40] Xu L., Chen Y., Feng A., Shi X., Feng Y., Yang Y., Wang Y., Wu Z., Zou Z., Ma W. (2023). Study on detection method of microplastics in farmland soil based on hyperspectral imaging technology. Environ. Res..

[41] Chen S., Ge L. (2019). Exploring the attention mechanism in LSTM-based Hong Kong stock price movement prediction. Quant. Financ..

[42] Long Q., Bagirov A., Taheri S., Sultanova N., Wu X. (2022). Methods and Applications of Clusterwise Linear Regression: A Survey and Comparison. ACM Trans. Knowl. Discov. Data.

[43] Cho H., Lee E.K. (2021). Tree-Structured Regression Model Using a Projection Pursuit Approach. Appl. Sci..

[44] Bentéjac C., Csörgő A., Martínez-Muñoz G. (2021). A comparative analysis of gradient boosting algorithms. Artif. Intell. Rev..

[45] Sricharoonratana M., Thompson A.K., Teerachaichayut S. (2021). Use of near infrared hyperspectral imaging as a nondestructive method of determining and classifying shelf life of cakes. LWT.

[46] Zhang Y., Zheng M., Zhu R., Ma R. (2022). Adulteration discrimination and analysis of fresh and frozen-thawed minced adulterated mutton using hyperspectral images combined with recurrence plot and convolutional neural network. Meat Sci..

[47] Thomas L.C. (2010). Consumer finance: Challenges for operational research. J. Oper. Res. Soc..

[48] Sharma S., Sumesh K.C., Sirisomboon P. (2022). Rapid ripening stage classification and dry matter prediction of durian pulp using a pushbroom near infrared hyperspectral imaging system. Measurement.

[49] Zhao W.Z., Du S.H. (2016). Spectral-Spatial Feature Extraction for Hyperspectral Image Classification: A Dimension Reduction and Deep Learning Approach. IEEE Trans. Geosci. Remote. Sens..

[50] Shin Y., Kim T., Hong S., Lee S., Lee E., Hong S., Lee C., Kim T., Park M.S., Park J. (2020). Prediction of Chlorophyll-aConcentrations in the Nakdong River Using Machine Learning Methods. Water.

[51] Sonobe R., Hirono Y., Oi A. (2020). Non-Destructive Detection of Tea Leaf Chlorophyll Content Using Hyperspectral Reflectance and Machine Learning Algorithms. Plants.

[52] De Amorim F.D.L., Rick J., Lohmann G., Wiltshire K.H. (2021). Evaluation of Machine Learning Predictions of a Highly Resolved Time Series of Chlorophyll-a Concentration. Appl. Sci..

[53] Narmilan A., Gonzalez F., Salgadoe A.S.A., Kumarasiri U., Weerasinghe H.A.S., Kulasekara B.R. (2022). Predicting Canopy Chlorophyll Content in Sugarcane Crops Using Machine Learning Algorithms and Spectral Vegetation Indices Derived from UAV Multispectral Imagery. Remote. Sens..

[54] Tang X.D., Huang M.T. (2022). Simulation of Chlorophyll a Concentration in Donghu Lake Assisted by Environmental Factors Based on Optimized SVM and Data Assimilation. Water.

