# Supplementary material for: Hyperspectral and Fluorescence Imaging Approaches for Nondestructive Detection of Rice Chlorophyll
Source: Plants (Basel). 2024 May 3;13(9):1270. doi: 10.3390/plants13091270 (PMC11085301; doi:10.3390/plants13091270)
Supplement: Supplementary file 1 [file plants-13-01270-s001.zip › plants-2830828-supplementary.pdf]

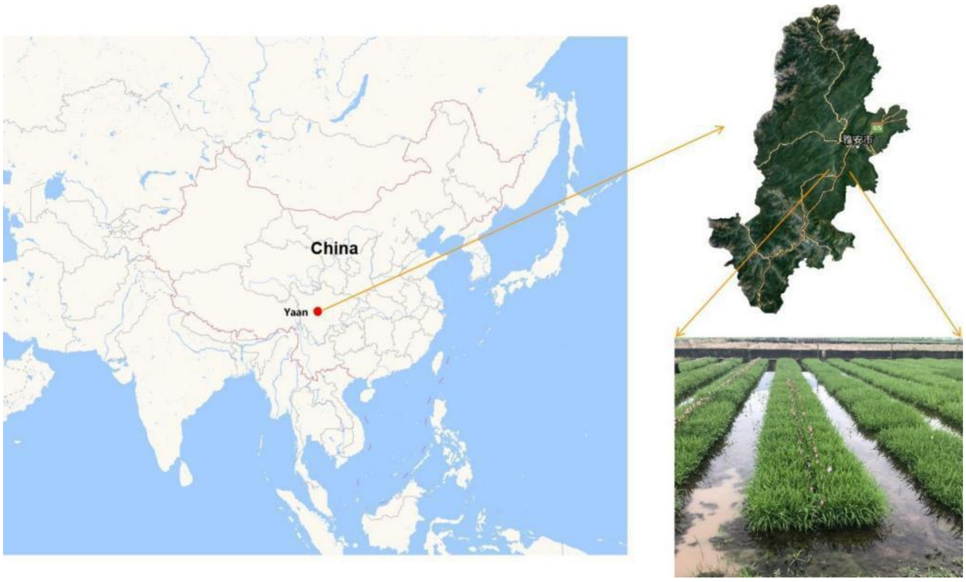

Figure S1. The location of the research area.

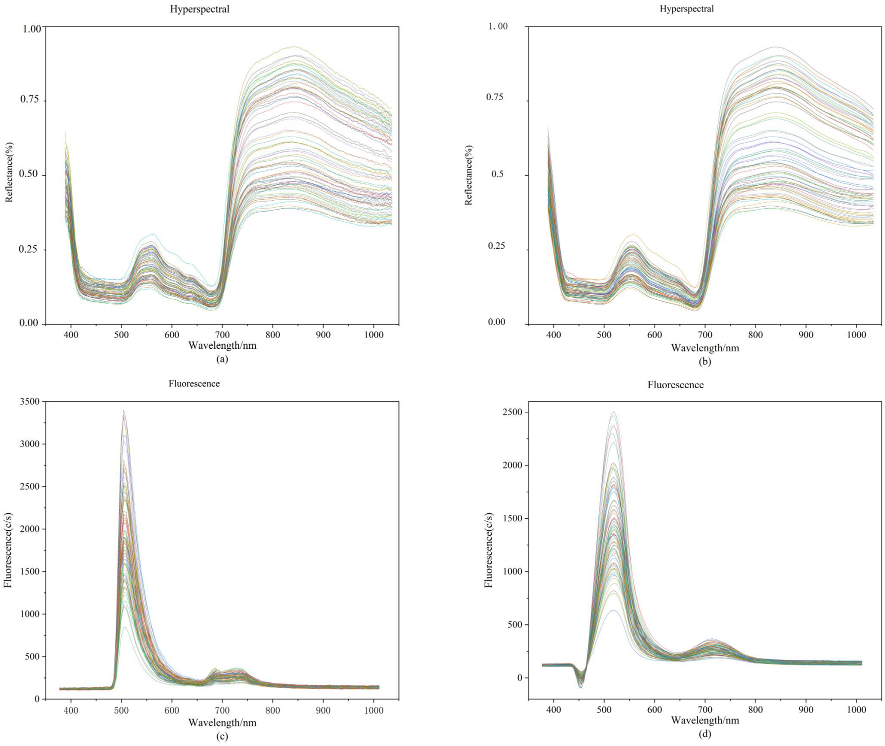

Figure S2. Comparison plot of data after SG convolution smoothing: (a) original hyperspectraldata, (b) hyperspectral data after convolution smoothing, (c) originalfluorescence spectral data, (d)fluorescence spectral data after convolution smoothing.

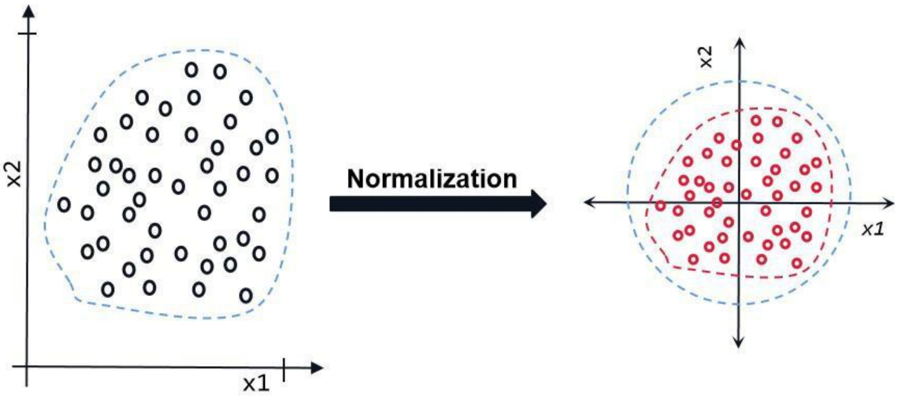

Figure S3. Schematic representation of the data normalization process.

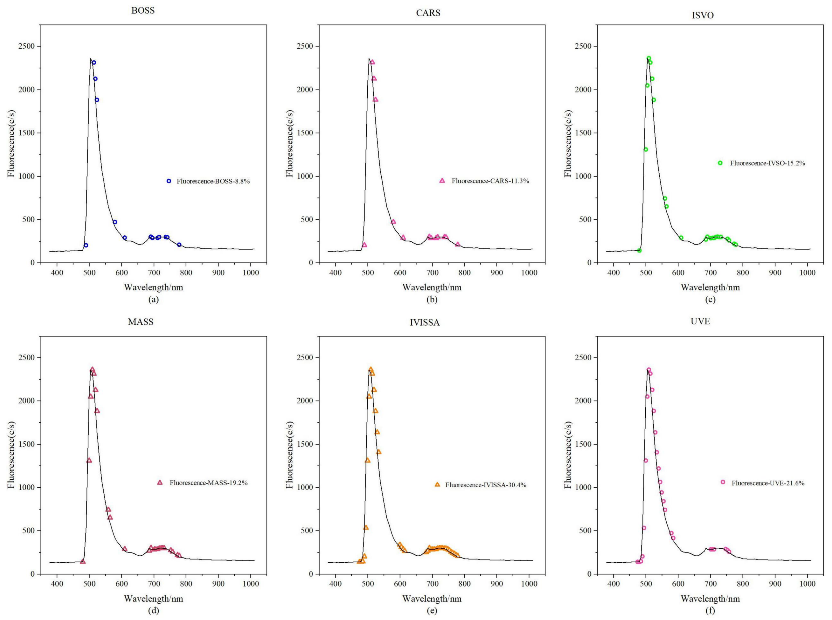

Figure S4. The illustration of the characteristic bands of the fluorescence spectrum obtained by different feature extraction algorithms.(a):Feature Extraction Results of BOSS Method; (b):Feature Extraction Results of CARS Method; (c):Feature Extraction Results of IVSO Method;(d):Feature Extraction Results of MASS Method;(e):Feature Extraction Results of IVISSA Method;(f):Feature Extraction Results of UVE Method.
